# Biochar Co-Applied with Lime Enhances Soil Phosphorus Availability via Microbial and Enzymatic Modulation of Paddy Soil

**DOI:** 10.3390/microorganisms13030582

**Published:** 2025-03-04

**Authors:** Yang Zhang, Caidi Yang, Jun Wang, Shenggao Lu

**Affiliations:** 1 Shaanxi Key Laboratory of Earth Surface System and Environmental Carrying Capacity, College of Urban and Environmental Science, Northwest University, Xi’an 710127, China; zy20185285@nwu.edu.cn (Y.Z.); yangcd@nwu.edu.cn (C.Y.); wangj@nwu.edu.cn (J.W.); 2 Shaanxi Key Laboratory for Carbon Neutral Technology, Northwest University, Xi’an 710127, China; 3 Zhejiang Provincial Key Laboratory of Agricultural Resources and Environment, College of Environmental and Resource Sciences, Zhejiang University, Hangzhou 310058, China

**Keywords:** P availability, P fractions, enzyme activity, P functional genes, microbial community structure

## Abstract

Soil microorganisms play a crucial role in improving soil phosphorus (P) availability. However, few studies have explored the changes in microbial community structure and their underlying mechanisms for improving soil P availability with the application of biochar and lime. Three kinds of biochar, made from rice straw (SB), Chinese fir wood sawdust (WB), and pig manure (MB), alone and with lime (SBL, WBL, and MBL), were applied to paddy soil to reveal the biochemical mechanisms for enhancing soil P availability. High-throughput sequencing and real-time PCR were used to investigate soil microbial communities and P functional genes. The three biochars increased the soil’s available P in the order of MB > SB > WB. Biochar co-applied with lime increased the available P (Olsen-P by 169–209%) and inorganic P (Al-P by 53.4–161%, Fe-P by 96.3–198%, and Ca-P by 59.0–154%) more than biochar alone, compared to the control (CK). Both biochar alone and co-applied with lime increased the activities of alkaline phosphomonoesterase (ALP), phosphodiesterase (PD), and inorganic pyrophosphatase (IPP) by 369–806%, 28.4–67.3%, and 37.9–181%, respectively, while it decreased the activity of acidic phosphomonoesterase (ACP) by 15.1–44.0%, compared to CK. Biochar, both alone and co-applied with lime, reduced the copy number of *phoC* gene by 5.37–88.7%, while it increased the *phoD*, *gcd*, and *pqqC* genes by 51.3–533%, 62.1–275%, and 25.2–158%, respectively, compared to CK. A correlation analysis and partial least squares path modeling (PLS-PM) indicated that Olsen-P, Bray-1 P, and inorganic P were significantly positively correlated with the activities of ALP, PD, IPP, and the *phoD* gene. Biochar co-applied with lime increased the relative abundances of the *phoD*-harboring bacteria *Proteobacteria*, *Firmicutes*, and *Acidobacteria*, which promoted the transformation of P to the effective state. Meanwhile, the dominant species *Anaerolinea*, *Ascomycota*, *Mucoromycota*, and *Chaetomium* provided rich effective nutrients for the soil microorganisms by accelerating the decomposition of soil organic matter, thus promoting phosphatase activity. It could be inferred that the optimized microbial community structure improved phosphatase activity by increasing the *phoD* gene and available nutrients, thus promoting the soil P availability. Biochar co-applied with lime had a better effect on increasing the P availability and rice yields than biochar alone.

## 1. Introduction

Phosphorus (P) deficiency is a global environmental problem affecting agricultural productivity [1]. To maintain an adequate available P level for crop production, a large amount of P fertilizer is applied to farmland soil [2]. However, due to the strong adsorption of orthophosphates by iron and aluminum oxides, the availability of P in soil remains low, especially in subtropical and tropical regions [3,4]. It is estimated that non-renewable phosphate rock used for the production of phosphate fertilizers will likely be exhausted this century [5]. Therefore, there is growing interest in the activation of organophosphorus and immobilized P in soil. Previous studies have shown that the addition of biochar can improve the soil P availability, with its efficacy influenced by the feedstock, pyrolysis temperature, dosage of biochar, and the soil type [6,7,8]. In addition, lime, a widely utilized amendment for ameliorating acidic soil, has proven effective in improving soil structure, nutrient availability, and crop yield [9,10].

In recent decades, considerable research efforts have been devoted to elucidating the chemical mechanism of biochar and lime for improving soil P availability [11,12,13]. The addition of lime has been demonstrated to significantly improve the availability of P for plants by increasing soil pH and reducing Al toxicity [14]. The chemical mechanisms by which biochar improves the soil P availability include direct P nutrient input, precipitation–dissolution transformations, and adsorption–desorption reactions [6,15]. In addition to these chemical transformations, the activation of organophosphorus, which accounts for 30–80% of the total P in soil, is the key to maintaining the sustainable utilization of available P [16,17]. Organophosphates in soil must undergo enzymatic hydrolysis before they can be utilized by plants and microorganisms [18]. The enzymes responsible for organophosphorus hydrolysis mainly include acid/alkaline phosphomonoesterase (ACP/ALP), phosphodiesterase (PD), and inorganic pyrophosphatase (IPP). Among these, ALP, encoded by the *phoA*, *phoD*, and *phoX* genes, plays a crucial role in converting organophosphorus into inorganic P, and the *phoD* gene has been widely detected in terrestrial ecosystems [19,20,21]. Khadem and Raiesi [22] found that maize straw biochar had a high potential to increase the activity of ALP, thereby promoting the mineralization and availability of organic P in arid soils. Yang and Lu [21] and Jin et al. [23] observed that biochar, derived from straw and manure, respectively, decreased ACP activity but increased ALP activity in soil. However, Zhou et al. [24] reported that the addition of woody biochar to forest soil did not enhance soil phosphatase activity. Gao and DeLuca [25] also found that biochar had no significant effect on the abundance of the *phoD* gene.

Generally, studies on the microbial mechanisms of biochar affecting the soil P cycle have mainly focused on the direct measurements of related enzyme activity and functional genes. However, there remains a critical gap in the understanding of the characterization of soil microbial community structures and their specific functions in this process [21,26]. At present, some *phoD*-harboring bacteria have been preliminarily screened, and these bacteria could promote the mineralization of organic P and the dissolution of inorganic P by improving enzyme activity to some extent, especially in P-deficient soils [27,28,29,30]. Hu et al. [31] found that an optimized inorganic–organic fertilizer application could improve the availability of P by regulating soil’s *phoD*-harboring bacteria community diversity and ALP activity. Tian et al. [32] found that the application of biochar under low-P conditions could optimize the *phoD* gene community, thereby promoting P mineralization, especially the specific enrichment of *Micromonosporaceae*. Liu et al. [33] applied rice husk biochar to three kinds of soils and found that biochar had the most significant effect on shaping the bacterial community structure in acidic red soil, significantly increasing the relative abundances of the soil-phosphate-solubilizing bacteria *Thiobacillus*, *Pseudomonas*, and *Flavobacterium* [27,33,34]. These findings suggested that the addition of biochar could change the microbial community structure and enhance the specific functions related to P cycling, ultimately promoting soil P availability. Furthermore, crop residue and manure biochar, characterized by a higher pH and specific surface area compared to lignocellulosic biochar, may promote the cycling of N and P more obviously by altering the microbial-mediated reactions [35,36]. Therefore, selecting biochar with different properties derived from straw, wood, and manure to explore its effects on soil P availability will help to fill the gap in understanding the microbial mechanisms underlying P availability improvement.

Although numerous studies have explored the microbial mechanisms by which biochar improves soil P availability, few studies have established the relationships among the soil microbial community with enzyme activity and P function genes, which constitutes a further novelty of the present study. Therefore, a pot experiment was carried out using the typical paddy soil treated with the biochar application alone and co-application with lime. The objectives were as follows: (1) examine the effects of biochar alone and co-applied with lime on the soil P availability, enzyme activity, and functional genes, (2) investigate the alterations in soil microbial community structure and functions related to soil P cycling, and (3) reveal the microbial mechanisms underlying the improvement in P availability. We hypothesize that (1) biochar co-applied with lime will improve soil P availability more effectively than biochar alone; (2) biochar alone and co-applied with lime will differently affect P-related enzyme activity, functional genes, and microbial community structure; and (3) specific enzymes, functional genes, and microbial communities will play important roles in P cycling and the improvement of P availability.

## 2. Materials and Methods

### 2.1. Experimental Materials

The experimental soil was collected from the surface layer (0–20 cm) of a paddy field located in Yueqing City, Zhejiang Province (28°08′ N, 120°87′ E). According to the Chinese Soil Taxonomy, this soil is classified as Stagnic Anthosol [37]. Following air-drying, the soil was sieved through a 2 mm mesh to prepare it for the pot experiment. The paddy soil exhibited the following basic properties: pH of 5.1, organic matter content of 20.58 g kg^−1^, cation exchange capacity (CEC) of 9.15 cmol kg^−1^, and clay content of 27.79 g kg^−1^. Lime was procured from a local agricultural market. Three agricultural wastes (rice straw, Chinese fir sawdust, and pig manure) were selected as feedstocks for biochar production. These materials were pyrolyzed at 550 ℃ for 2 hours to yield straw biochar (SB), woody biochar (WB), and pig manure biochar (MB), respectively. The pH and elemental composition of both the lime and the three biochars are detailed in Table 1. The pH was quantified using a pH meter with a 1:20 ratio of biochar/lime to deionized water. The elemental composition was determined by atomic fluorescence spectrophotometer (AFS-9760, HaiGuang Instrument Co., Ltd., Beijing, China).

### 2.2. Pot Experiment and Soil Sampling

The pot experiment was conducted using 5 kg of dry soil per pot, with each pot measuring 32 cm in diameter and 20.9 cm in height. Seven treatments were established: lime application (0.5 g kg^−1^, labeled as Lime), individual biochar application (5 g kg^−1^, labeled as SB, WB, and MB for straw biochar, woody biochar, and pig manure biochar, respectively), and co-application of biochar with lime (5 g kg^−1^ biochar+0.5 g kg^−1^ lime, labeled as SBL, WBL, and MBL). The lime and biochar were uniformly mixed into the soil. A control treatment (CK) without lime and biochar was also included. Each treatment was replicated three times.

Following a 30-day incubation period, rice seedlings (cultivar Zhongzheyou 8) were transplanted into pots in March 2021, aligning with the standard practices of field rice production. Uniform irrigation, transplantation, fertilization, and water management protocols were applied across all treatments. A base fertilizer of 0.15 g kg^−1^ rice compound fertilizer was applied, supplemented with 0.05 g kg^−1^ rice compound fertilizer and 0.05 g kg^−1^ urea as top-dressing. Rice was harvested upon maturation in July 2021, with grains and above-ground biomass weighed separately.

Soil samples were collected at a distance of 5 cm from the rice roots using a soil sampler. Three sub-samples from each pot were combined into a composite sample, which was then divided into two portions. One portion was air-dried, crushed, and sieved through 2 mm and 0.15 mm meshes for chemical property analysis. The other portion was stored at −80 ℃ for subsequent soil microbial analysis. The basic properties of the soils amended with biochar and lime are summarized in Table 2.

### 2.3. Soil Biochemical Analysis

Soil pH was measured using a pH meter with a soil-to-deionized water ratio of 1:2.5 [38]. The exchangeable acid content was determined by the KCl extraction and NaOH titration [38]. Soil organic matter (SOM) content was determined by the potassium dichromate oxidation method [38]. Cation exchange capacity (CEC) was measured by the NH_4_OAc extraction procedure [38]. Available potassium (K) was measured using a flame photometer [38]. Total P was extracted using the NaOH melting method, while Olsen-P, Bray-1 P, water-extractable P (H_2_O-P), and sulfuric acid-extractable P (H_2_SO_4_-P) were extracted using NaHCO_3_, HCl + NH_4_F, deionized water, and HCl + H_2_SO_4_, respectively [38]. The P concentration in the extraction solutions was determined by the molybdenum–antimony colorimetric method [39].

Soil microbial biomass carbon (MBC), nitrogen (MBN), and P (MBP) were extracted using the chloroform fumigation extraction method [40]. The contents of MBC and MBN were directly analyzed using a carbon and nitrogen analyzer, while MBP was measured using the molybdenum–antimony colorimetric method [39]. Dehydrogenase activity (DHA) was determined based on the reduction of triphenyltetrazolium chloride (TTC) to triphenylformazan (TPF) [41]. The activities of acidic phosphomonoesterase (ACP), alkaline phosphomonoesterase (ALP), phosphodiesterase (PD), and inorganic pyrophosphatase (IPP) were determined by the *p*-nitrophenyl phosphate method, 4-nitrophenyl phosphate disodium salt hexahydrate method, and sodium pyrophosphate colorimetric method, respectively [41].

### 2.4. Identification of Microbial Communities and Genes

Total DNA was extracted from 0.5 g of fresh samples employing the FastDNA SPIN Kit (MP Bio-medicals, Solon, OH, USA) according to the manufacturer’s instructions. The concentration and quality of the extracted DNA were assessed using a Nanodrop ND-1000 spectrophotometer (NanoDrop Technologies, Wilmington, DE, USA). Amplification of the V4-V5 region of the 16S rRNA gene was performed using the universal primers 515F (GTGCCAGCMGCCGCGGTAA) and 907R (CCGTCAATTCCTTTGAGTTT), and the fungal ITS1 gene region was amplified using the primers ITS5-1737F (GGAAGTAAAAGTCGTAACAAGG) and ITS2-2043R (GCTGCGTTCTTCATCGATGC) [42]. The PCR products were sequenced using an Illumina HiSeq platform following the standard protocols: 94 °C for 5 min, 30 cycles at 94 °C for 30 s, 52 °C for 30 s, 72 °C for 30 s, a final 10 min extension at 72 °C, and then collecting data after annealing at 4 °C. Raw sequence data generated by base calling were stored in FastQ format. Data preprocessing included filtering of paired-end raw reads, assembly of paired-end clean reads, and quality filtering of raw tags. Operational taxonomic units (OTUs) were clustered at a 97% similarity threshold using the UPARSE method.

DNA extracts were also utilized for the amplification of P-related functional genes. Real-time quantitative PCR (qPCR) was conducted using the GoTaq^®^ qPCR Master Mix kit (Promega, Madison, USA) on an ABI 7300 Cycle Real-time PCR System (Applied Biosystems, Darmstadt, Germany). The qPCR protocols were comprised of 95 °C for 2 min, 40 cycles at 95 °C for 15 s, 55 °C or 30 s, 72 °C for 30 s, and then collecting data after annealing at 52 °C. The primer sequences and detailed experimental procedures were adapted from the studies of Yang and Lu [21] and Fraser et al. [43,44]. All sequencing and amplification processes were carried out by Magigene Co., Ltd. (Shenzhen, China). The raw sequencing reads have been deposited in the National Center for Biotechnology Information (NCBI) Sequence Read Archive (SRA) under the BioProject accession number PRJNA822008.

### 2.5. Statistical Analyses

Statistical analyses were performed using SPSS 17.0 (SPSS, Inc., Chicago, IL, USA). A one-way analysis of variance (ANOVA) was carried out to determine significant differences between treatments using the LSD test with a significant level of *p* < 0.05. A two-way analysis of variance (ANOVA) was used to examine the contribution of biochar type (BT) and whether biochar was applied with lime (BL) on the soil properties. To examine the relationships between microbial community structure and P-related enzyme activities, as well as functional genes, co-occurrence networks were constructed based on Pearson’s correlation coefficients with statistical significance at *p* < 0.05 (Bonferroni-corrected). Gephi software (version 0.9.2) was used to visualize the networks [45]. The partial least squares path modeling (PLS-PM) was performed using the “plspm” package to elucidate the possible pathways of P conversion influenced by the soil physicochemical properties and microbial community structure [46].

## 3. Results

### 3.1. Grain Yield and Straw Biomass of Rice

**Figure 1:**
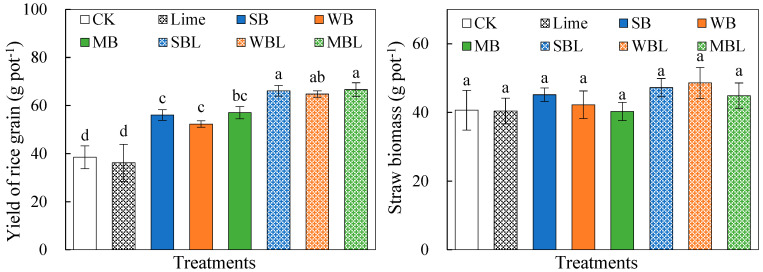
Effects of biochar application alone and co-application with lime on rice grain yield and straw biomass. Error bars represent the standard error of the means (*n* = 3). Different letters above error bars indicate significant differences between treatments (*p* < 0.05, LSD test).

The effects of biochar application alone and co-application with lime on rice grain yield and straw biomass are shown in Figure 1. Compared to CK, biochar application alone and co-application with lime significantly (*p* < 0.05) increased the rice grain yield by 36.0–48.2% and 68.4–73.1%, which reached the highest increase with the MB and MBL treatments, respectively. Regarding applying biochar alone or co-applying with lime, the effect of the three biochars on the rice yield showed no significant differences. However, none of the treatments had significant effects on the straw biomass.

### 3.2. Availability and Forms of P in Soil

**Figure 2:**
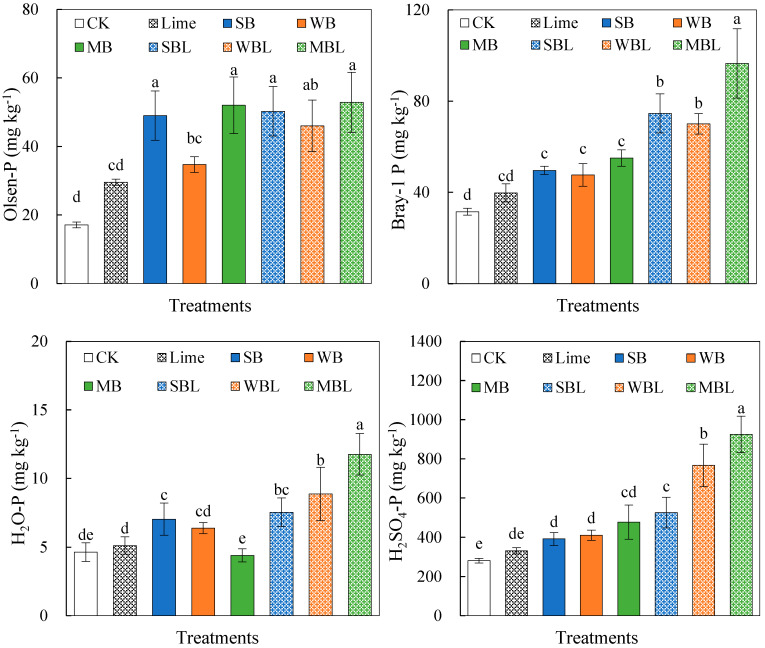
Effects of biochar application alone and co-application with lime on the contents of soil Olsen-P, Bray-1 P, H_2_O-P, and H_2_SO_4_-P. Error bars represent the standard error of the means (*n* = 3). Different letters above error bars indicate significant differences between treatments (*p* < 0.05, LSD test).

The effects of biochar application alone and co-application with lime on the soil P availability are shown in Figure 2. Both biochar application alone and co-application with lime significantly (*p* < 0.05) increased the soil Olsen-P content. The applications of MB, WB, and SB increased the Olsen-P by 186%, 103%, and 204%, respectively, while their co-applications with lime increased the Olsen-P by 194%, 169%, and 209%, respectively. However, no statistically significant differences were observed in the Olsen-P content between biochar alone and co-application with lime (*p* > 0.05). Both biochar application alone and co-application with lime significantly (*p* < 0.05) increased the Bray-1 P content compared to CK. Notably, biochar co-applied with lime had a higher Bray-1 P content than the biochar alone, which exhibited increases of 163%, 147%, and 240% in SBL, WBL, and MBL treatments, respectively. Both H_2_O-P and H_2_SO_4_-P with biochar co-applied with lime were higher than those with biochar alone. The MBL treatment increased H_2_O-P and H_2_SO_4_-P by 153% and 229%, respectively, compared to CK.

**Figure 3:**
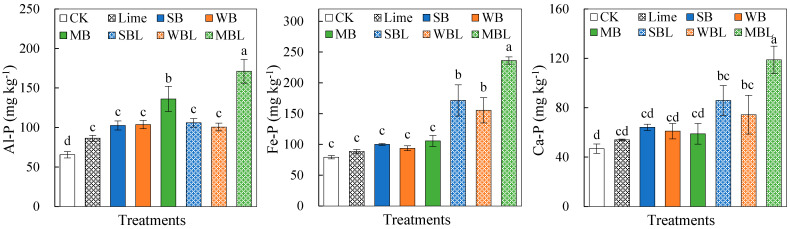
Effects of biochar application alone and co-application with lime on chemical fractional P in soil. Error bars represent the standard error of the means (*n* = 3). Different letters above error bars indicate significant differences between treatments (*p* < 0.05, LSD test).

The effects of biochar alone and co-applied with lime on the contents of soil inorganic P are shown in Figure 3. Soils amended with biochar alone and co-applied with lime had a significantly higher Al-P content than CK, which increased by 108% and 161% in the MB and MBL treatments, respectively. Biochar application alone had no significant effects on the soil Fe-P, but biochar co-applied with lime increased the Fe-P by 117%, 96.3%, and 198%, respectively. Only biochar co-applied with lime significantly (*p* < 0.05) increased the Ca-P content, which increased by 83.6%, 59.0%, and 154% in the SBL, WBL, and MBL treatments, respectively.

### 3.3. Soil Enzyme Activities and P Functional Genes

The effects of biochar application alone and co-application with lime on soil P-related enzyme activities are shown in Table 3. Compared to CK, the activity of DHA significantly (*p* < 0.05) decreased by 15.3%, 28.7%, and 47.8% in SB, MB, and MBL, respectively. Both biochar application alone and co-application with lime significantly (*p* < 0.05) reduced ACP activity by 15.1–44.0%, whereas it increased the activities of ALP, PD, and IPP by 369–806%, 28.4–67.3%, and 37.9–181%, respectively. Notably, MBL treatment demonstrated the largest increase in ALP and IPP activities. The MB, SBL and MBL treatments had higher PD activity than the other treatments. A two-way ANOVA showed that the biochar type had more obvious effects on soil enzyme activity, and whether biochar was applied with lime only had significant effects on the activities of ACP, ALP, and IPP (Table 3).

Table 4 shows the alterations of soil P functional genes under different treatments. Lime application alone significantly reduced the copy numbers of the *phoC*, *gcd*, and *pqqC* genes by 95.2%, 66.5%, and 78.6%, respectively, compared to CK. Both biochar application alone and co-application with lime reduced the copy number of the *phoC* gene by 5.37–88.7% while it increased the *phoD*, *gcd*, and *pqqC* genes by 51.3–533%, 62.1–275%, and 25.2–158%, respectively, compared to CK. Compared to biochar alone, the co-application of biochar with lime resulted in a greater reduction in the copy number of the *phoC* gene and a greater increase in the copy number of the *phoD* gene, respectively. Whether biochar was applied alone or with lime, MB had the largest copy numbers of the *gcd* and *pqqC* genes among the three biochars. A two-way ANOVA demonstrated that both the biochar type and whether the biochar was applied with lime had significant effects on the soil P functional genes *phoC*, *phoD*, *gcd*, and *pqqC* (Table 4).

### 3.4. Soil Microbial Community Structure

**Figure 4:**
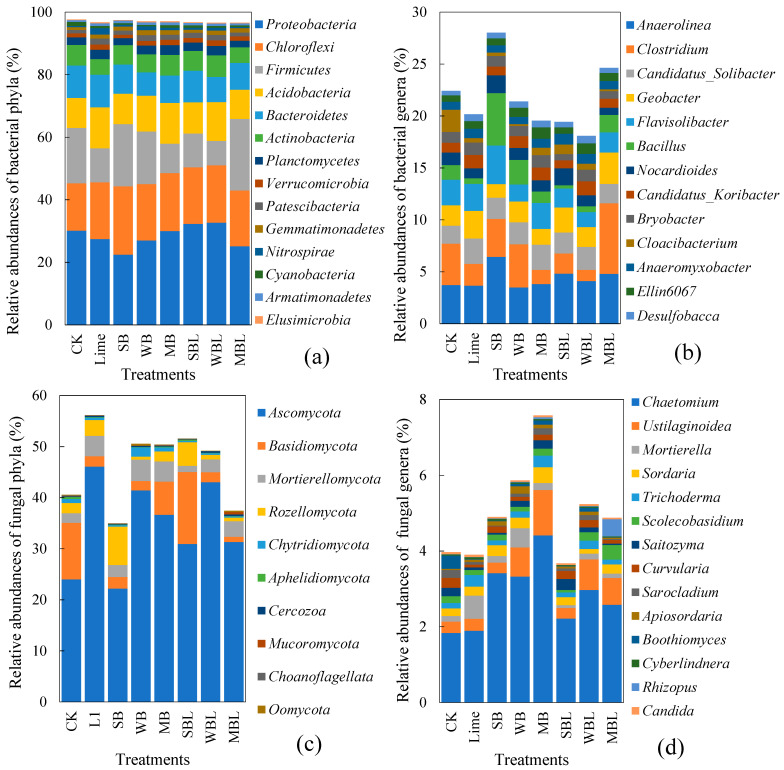
Effects of biochar application alone and co-application with lime on soil microbial community structure. (**a**) Bacterial phylum level, (**b**) bacterial genus level, (**c**) fungal phylum level, (**d**) fungal genus level.

The alterations in soil microbial community structure with the application of biochar and lime are shown in Figure 4. For the bacterial phyla with relative abundances exceeding 0.1% (Figure 4a), both biochar alone and co-applied with lime increased the relative abundances of *Chloroflexi*, *Acidobacteria*, *Patescibacteria*, *Gemmatimonadetes*, *Nitrospirae*, and *Armatimonadetes,* while decreasing those of *Bacteroidetes*, *Actinobacteria*, *Planctomycetes*, and *Cyanobacteria*. Notably, SBL and WBL significantly (*p* < 0.05) increased the relative abundances of the dominant bacterial phylum *Proteobacteria*, while MBL increased that of *Firmicutes*. The biochar type largely affected the relative abundances of bacterial genera (Figure 4b). Both biochar alone and co-applied with lime significantly (*p* < 0.05) increased the relative abundances of *Anaeromyxobacter*, *Ellin6067*, and *Desulfobacca* while decreased *Candidatus_Koribacter* and *Cloacibacterium*. Among the treatments, the SBL treatment had the largest increase in the relative abundances of key bacteria genera, including *Anaerolinea*, *Flavisolibacter*, *Bacillus*, and *Nocardioides*, while the MBL treatment increased the relative abundances of *Clostridium* and *Geobacter* more obviously.

The fungal phyla communities (Figure 4c) showed that both biochar application alone and co-application with lime generally increased the relative abundances of *Ascomycota* and *Mortierellomycota,* while decreasing those of *Aphelidiomycota* and *Choanoflagellata*. In addition, the SBL treatment significantly (*p* < 0.05) increased the relative abundances of *Basidiomycota* and *Rozellomycota*, while MBL increased those of *Cercozoa*, *Mucoromycota*, and *Oomycota*. At the fungal genus level, *Chaetomium* accounted for the largest proportion (Figure 4d). All treatments significantly (*p* < 0.05) increased the relative abundance of *Chaetomium*, but biochar alone had a larger increase than biochar co-applied with lime. Three kinds of biochar also increased the relative abundances of *Sordaria*, *Apiosordaria*, *Cyberlindnera*, and *Rhizopus*. The WB, MB, WBL, and MBL treatments significantly increased the relative abundance of *Ustilaginoidea* in soil, compared to the SB and SBL treatments. The MBL treatment exhibited the largest relative abundances of *Scolecobasidium* and *Rhizopus*.

### 3.5. Correlations of Chemical and Microbial Properties with Availability and Forms of P in Soil

**Figure 5:**
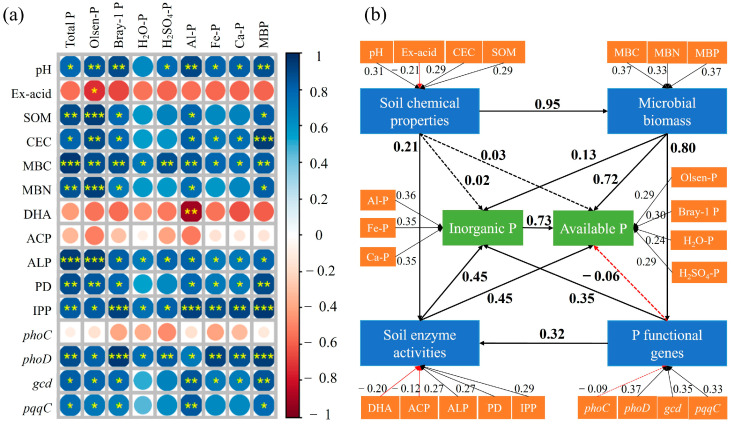
Pearson correlation analysis of soil chemical properties, enzyme activities, and P functional genes with P availability and forms (**a**) and the directed graph of the partial least squares path model (PLS-PM) (Gof = 0.841) (**b**). (**a**) Red and blue circles represent negative and positive correlations, respectively. Circle sizes from large to small and color changes from dark to light indicate the correlations from high to low. “*” in circles indicates significance between the chemical or microbial properties and P availability or forms (*, *p* < 0.05; **, *p* < 0.01; ***, *p* < 0.001). (**b**) Each box represents an observed variable. The green boxes represent the inorganic and available P. The blue boxes represent the chemical and microbial properties (microbial biomass, enzyme activities, and P functional genes). The orange boxes represent the latent variables. The black and red arrows represent positive and negative effects, respectively. Solid and dashed lines indicate significant (*p* < 0.05) and non-significant (*p* > 0.05) correlations, respectively.

Figure 5a illustrates the direct correlations between soil chemical and microbial properties and P availability and forms. Soil pH, SOM, and CEC exhibited significant positive correlations with Olsen-P and Bray-1 P. Soil pH was also positively correlated with Al-P (R = 0.90, *p* < 0.01), Fe-P (R = 0.81, *p* < 0.05), Ca-P (R = 0.82, *p* < 0.05), and MBP (R = 0.82, *p* < 0.01). For the soil microbial properties, the activities of ALP, PD, and IPP showed strong positive correlations with the available and inorganic P. Olsen-P was correlated with the activities of ALP (R = 0.94, *p* < 0.001), PD (R = 0.85, *p* < 0.01), and IPP (R = 0.94, *p* < 0.001). The activity of IPP showed more significant correlations with Al-P, Fe-P, Ca-P, and MBP. Additionally, P-related functional genes, including *phoD*, *gcd*, and *pqqC*, displayed strong positive correlations with P availability and forms, especially the *phoD* gene. Bray-1 P was positively correlated with the *phoD* gene (R = 0.94, *p* < 0.001), and Al-P showed positive correlations with the *gcd* gene (R = 0.86, *p* < 0.01) and *pqqC* gene (R = 0.85, *p* < 0.01), respectively. To better integrate these complex interrelationships, a PLS-PM was constructed (Figure 5b). Soil chemical properties mainly affected the P availability and forms by affecting microbial activities, such as the enzyme activities (0.21) and microbial biomass (0.95). The microbial properties showed direct effects on the P availability and forms. Soil enzyme activities showed positive direct effects on inorganic P (0.45) and available P (0.45). The P-related functional genes showed positive direct effects on the inorganic P (0.35).

### 3.6. Relationship of Dominant Microbial Community with Enzyme Activity and Functional Genes

**Figure 6:**
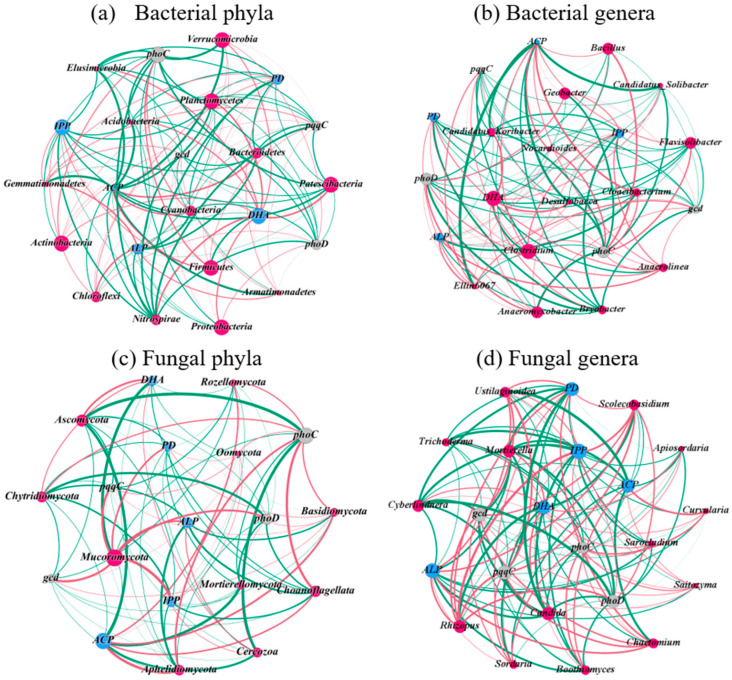
Co-occurrence network analysis of dominant soil microbial community with enzyme activities and functional genes based on correlation analysis. A red edge indicates a positive relationship, while a green edge indicates a negative relationship between two individual nodes. The red, blue, and gray nodes represent the dominant microorganism species, enzyme activities, and functional genes, respectively. The thickness of each connection between two nodes (i.e., edge) is proportional to the value of Spearman’s correlation coefficients.

Figure 6 shows the co-occurrence network analysis of dominant microbial communities with soil enzyme activities and functional genes. For the bacterial phylum (Figure 6a), the DHA activity was significantly (*p* < 0.05) positively correlated with *Patescibacteria* (0.61), *Nitrospirae* (0.56), and *Planctomycetes* (0.56). The ALP activity was significantly (*p* < 0.05) positively correlated with *Chloroflexi* (0.56) and *Gemmatimonadetes* (0.52). The ACP activity was significantly (*p* < 0.05) correlated with *Bacteroidetes* (0.61) and *Cyanobacteria* (0.51). For the bacterial genus (Figure 6b), the dominant *Desulfobacca* and *Candidatu_Koribacter* showed significant positive correlations with DHA activity, with coefficients of 0.55 and 0.54, respectively. ACP activity was significantly (*p* < 0.05) positively correlated with *Cloacibacterium* (0.63), while ALP activity was correlated with *Anaerolinea* (0.50). The *phoC* gene was significantly (*p* < 0.05) positively correlated with *Nocardioides* (0.52) and *Bacillus* (0.61), while the *phoD* gene was positively correlated with *Anaeromyxobacter* (0.53).

For the fungal phylum (Figure 6c), the dominant *Mucoromycota* was positively correlated with IPP activity and the copy numbers of the *phoC*, *phoD*, and *gcd* genes, with respective coefficients of 0.77, 0.62, 0.58, and 0.55. *Ascomycota* showed a positive correlation with DHA activity (R = 0.52, *p* < 0.05) but a negative correlation with ACP activity (R = −0.71, *p* < 0.05). For the fungal genus (Figure 6d), *Rhizopus* had significant (*p* < 0.05) positive correlations with IPP activity, ALP activity, and the copy numbers of the *pqqC*, *gcd* and *phoD* genes, with coefficients of 0.79, 0.62, 0.59, 0.58, and 0.53, respectively. *Scolecobasidium* was positively correlated with IPP activity, ALP activity, and the *pqqC* gene, with coefficients of 0.59, 0.52, and 0.50, respectively. *Chaetomium* was significantly (*p* < 0.05) positively correlated with ALP activity (0.57) and the *pqqC* gene (0.53). *Ustilaginoidea* was positively correlated with the *pqqC* and *gcd* genes, with coefficients of 0.63 and 0.56, respectively.

## 4. Discussion

### 4.1. Effects of Biochar Application Alone and Co-Application with Lime on Soil P Availability and Forms

The results indicated that biochar co-applied with lime had a greater increase in the available P than biochar alone, and the three kinds of biochar increased the available P in the order of MB > SB > WB. Firstly, the application of biochar provided direct P input to the soil through its inherent P content. Among the three kinds of biochar, MB contained the highest P content (Table 1), which consequently led to the largest increase in available P when MB was applied. Secondly, the combined application of biochar and lime largely modified the soil chemical properties, thereby influencing the conversion of P in the soil [14,47]. The increased soil pH facilitated the conversion of insoluble phosphate into more soluble forms. Although the application of biochar alone could reduce the adsorption of phosphate by increasing the soil pH and the precipitation of Al^3+^ and Fe^3+^ [48], co-application with lime increased the soil pH much more than biochar alone (Table 1). This enhanced pH effect explained the superior performance of the biochar–lime co-application in increasing the soil available P. Correlation analysis also showed that the soil pH was significantly positively correlated with the Olsen-P and Bray-1 P (Figure 5a).

In acidic soils, the majority of phosphates were immobilized with free metal ions to form minerals or adsorbed by soil minerals (Fe and Al oxides) [15]. Therefore, the content and conversion of soil inorganic P had crucial effects on the available P [49]. The fractional P content in the studied soil followed the order of Fe-P > Al-P > Ca-P, which aligned with previous studies on acidic soils [21,50]. Both biochar application alone and co-application with lime significantly increased the soil Al-P content, while the co-application of biochar with lime increased the Fe-P and Ca-P contents. The metal elements (Fe and Al) in biochar ash could form Fe and Al oxides that adsorbed phosphate, thereby increasing the Al-P and Fe-P. However, the addition of Ca from lime contributed to the elevation of Ca-P [51]. Notably, due to the fewer P adsorption sites in Al compounds than Fe compounds, Al-P became the primary contributor to the soil available P pool as soil pH increased [52]. Only at the higher pH could the large amount of OH^−^ promote the desorption and dissolution of Fe-P, subsequently increasing the soil available P [53]. PLS-PM showed that Al-P had a greater effect on the soil available P than Fe-P and Ca-P (Figure 5b). Correlation analysis indicated that the soil Al-P, Fe-P, and Ca-P had significant positive correlations with pH, as well as with the activities of ALP, PD, and IPP (Figure 5a). Therefore, the addition of biochar and lime enhanced the inorganic P not only by increasing the soil pH, but also by increasing the soil enzyme activity, especially the co-application of biochar with lime. The porous structure and high nutrient content of biochar provided favorable conditions for soil microorganisms, thereby promoting the activity of P-mineralizing enzymes [54].

### 4.2. Effects of Biochar Application Alone and Co-Application with Lime on Soil Enzyme Activities and P Functional Genes

The effects of biochar alone and co-applied with lime on the P availability were closely related to the microbial activities in soils. Soil dehydrogenase (DHA), a redox enzyme, served as an indicator of the intensity and direction of soil biochemical processes and was closely related to soil fertility [55]. However, the application of biochar and lime decreased or had no significant effect on DHA activity. Although the active organic and volatile substances from biochar could stimulate DHA activity, DHA activity tended to be inhibited under high-P conditions [56]. Soil phosphatase played a key role in the mineralization of soil organophosphates [57]. ACP was primarily derived from plant roots while ALP was produced by soil microorganisms, and both enzymes facilitated the transformation of soil organic P to inorganic P [58]. The product of soil ACP, ALP, and IPP was active P that plants could directly absorb and utilize, while phosphodiesterase (PD) only hydrolyzed phosphodiester to produce phosphomonoester rather than orthophosphate. Therefore, the sequential action of these phosphatases eventually led to the release of free phosphate [27]. The addition of biochar and lime significantly increased the activities of ALP, PD, and IPP while the ACP activity decreased, which was consistent with previous results [21]. Jain et al. [59] found that the direct increase in the soil available P from biochar water-soluble P reduced Fe^3+^ that could catalyze phosphatase reactions, thus reducing ACP activity. Additionally, the optimal pH for ACP activity was 4.0, while ALP activity peaked at pH 10, suggesting that the application of biochar and lime modulated the enzyme activity by altering the soil pH [60,61]. As a key enzyme in soil organic P mineralization, the ALP activity was influenced by its coenzyme factors Mg^2+^ and Zn^2+^ [62]. The activities of ALP and PD were affected by the soil P availability, increasing only under low-P conditions, which could be explained by the resource allocation theory [32,63]. Furthermore, soil phosphatase activities were regulated by the related functional genes. The application of biochar alone and with lime significantly increased the copy numbers of the *phoD* and *gcd* genes while decreasing *phoC*. The *phoC* and *phoD* genes played a key role in regulating ACP and ALP activities, respectively, thereby promoting soil organophosphorus mineralization. However, the *gcd* and *pqqC* genes primarily promoted soil inorganic P solubilization [25,64]. Correlation analysis further showed a positive correlation between the copy numbers of the *phoD* gene and the available P across different extraction methods, as well as the inorganic P (Figure 5a).

### 4.3. The Changes in Soil Microbial Community Structure and Functions Improving P Availability

**Figure 7:**
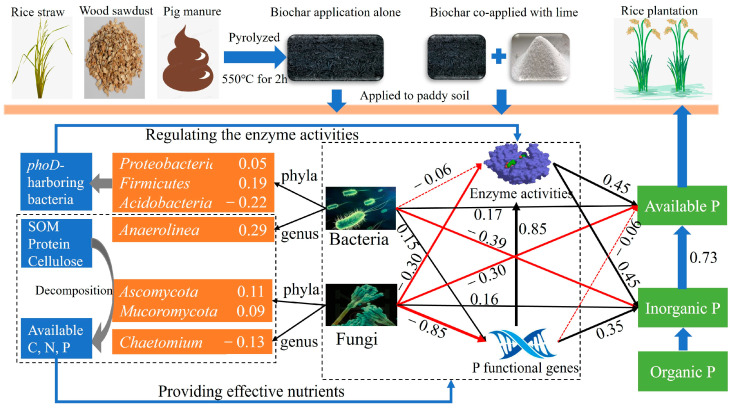
Diagram illustrating the microbial mechanisms of P conversion and utilization in soil based on the partial least squares path models (PLS-PM) (Gof = 0.578). The blue arrows represent the transformation or action processes. The black and red arrows represent positive and negative effects, respectively. Solid and dashed lines indicate significant (*p* < 0.05) and non-significant (*p* > 0.05) correlations, respectively.

The dominant bacterial phyla in the paddy soil were *Proteobacteria*, *Chloroflexi*, *Firmicutes*, *Acidobacteria*, *Bacteroidetes*, and *Actinobacteria*. Among them, *Proteobacteria*, *Firmicutes*, and *Acidobacteria* were the active *phoD*-harboring bacteria that regulated ALP activity, thereby increasing the soil P availability [29,30]. *Bacteroidetes* was an important *phoC*-harboring bacterial taxon, whose declining relative abundance reduced ACP activity [65,66]. Correlation analysis also showed a significant positive correlation between ACP activity and *Bacteroidetes* abundance. The dominant bacterial genera were *Anaerolinea*, *Clostridium*, *Candidatus_Koribacter*, *Geobacter*, *Flavisolibacter*, and *Bacillus*. *Anaerolinea* abundance was significantly positively correlated with ALP activity (R = 0.50, *p* < 0.05). As a representative genus of the *Chloroflexi* phylum, *Anaerolinea* played a crucial role in pig manure degradation during anaerobic digestion, accelerating soil organic matter decomposition and thereby providing nutrients for soil microbes [67]. The anaerobic characteristics of the rice paddy field created favorable conditions for *Chloroflexi* species [68]. Collectively, the bacterial phyla *Proteobacteria*, *Chloroflexi*, *Firmicutes*, and *Acidobacteria* and the genus *Anaerolinea* promoted P availability by regulating P mineralization enzymes and creating suitable nutrient environments (Figure 7).

The dominant fungal phyla in the paddy soil were *Ascomycota*, *Choanoflagellata*, *Mortierellomycota*, and *Rozellomycota*. Both biochar application alone and co-application with lime increased the relative abundances of *Ascomycota* and *Mortierellomycota*. There was a significant positive correlation between the abundance of *Ascomycota* and DHA activity, indicating that *Ascomycota*, which accounted for the majority of the total fungal community (about 30%), played a crucial role in mediating soil redox processes (Figure 6c). In addition, *Ascomycota* existed in soil with nutritional routes that were saprophytic, parasitic, and symbiotic and participated in the decomposition of SOM [69]. The MBL treatment significantly increased the relative abundance of *Mucoromycota*, which was significantly positively correlated with IPP activity and the copy numbers of the *phoC*, *phoD*, and *gcd* genes (Figure 6c). The increased abundance of *Ascomycota* and *Mucoromycota* could promote the decomposition of soil organic matter and proteins, respectively, thus providing effective nutrients for microbial growth and reproduction [69,70]. In addition, the increased relative abundance of the fungal genus *Chaetomium* could provide suitable living conditions for soil microorganisms by the decomposition of cellulose (Figure 7). Correlation analysis also showed a positive correlation between the *Chaetomium* abundance and the ALP activity, which was consistent with the results of Huang et al. [27] reporting a positive correlation between *Chaetomium* and the *phoD* gene.

### 4.4. Microbial Mechanisms of Biochar and Lime Improving P Availability in Paddy Soils

Based on the PLS-PM, a concept framework elucidating the microbial mechanisms of P conversion and utilization was proposed in Figure 7. The main ways to increase the soil available P were the enzymatic hydrolysis of organic P and the solubilization of inorganic P. The enzymatic hydrolysis of organic P was driven by various phosphatases including ACP, ALP, PD, and IPP [43]. These phosphatases, encoded by the specific genes, were related to the community structure of soil bacteria and fungi [28]. Therefore, the microbial mechanisms of biochar application alone and co-application with lime in improving soil P availability are summarized as follows. Firstly, biochar provided favorable conditions and essential nutrients for the survival and reproduction of soil microbes involved in P cycling. Secondly, the enhancement of soil microbial activity (such as that of *Anaerolinea*, *Ascomycota*, *Mucoromycota*, and *Chaetomium*) promoted the decomposition of SOM, proteins, and cellulose, which subsequently released bioavailable nutrients for microbial utilization (Figure 7). Thirdly, the increased relative abundances of *phoD*-harboring bacterial phyla (*Proteobacteria*, *Firmicutes*, and *Acidobacteria*) promoted the mineralization of P (Figure 7). On the one hand, the co-application of biochar with lime increased the soil available P content more than biochar alone by improving the soil biochemical properties. On the other hand, the greater increased activity of P-related enzymes and microbial communities in MB- and SB-treated soils could lead to a higher available P content. Therefore, both the biochar type and application method should be considered to improve soil P availability in the farmland ecosystem. Notably, the phosphatase was the key enzyme promoting the mineralization of soil organic P. Its activity was affected not only by soil biochemical properties, but also by the related coenzyme factors. This insight provided valuable direction for further study into the microbial mechanisms underlying soil P cycling and offered potential pathways for enhancing P management in agricultural systems.

## 5. Conclusions

The co-application of biochar with lime increased the soil available P and inorganic P more than biochar alone. Among three kinds of biochar, MB had the greatest increase in the soil available P. The application of biochar alone significantly increased the Al-P content while biochar co-applied with lime increased the Al-P, Fe-P, and Ca-P contents. In addition to the direct input and chemical conversion of phosphate, the alteration of microbial activity and community structure played a crucial role in increasing the soil P availability. The increased activities of ALP, PD, and IPP and the copy numbers of the *phoD* and *gcd* genes promoted the mineralization of soil organic P, especially under biochar co-applied with lime. In addition, biochar application increased the relative abundances of the active *phoD*-harboring bacteria *Proteobacteria*, *Firmicutes*, and *Acidobacteria*, thereby directly promoting phosphatase activity. The bacterial genus *Anaerolinea*, dominant fungal phyla *Ascomycota* and *Mucoromycota*, and fungal genus *Chaetomium* could promote the phosphatase activities by providing plenty of effective nutrients. Therefore, the increased soil phosphatase activity and optimized specific microbial communities collectively improved the P availability, with the co-application of biochar with lime showing superior efficacy compared to biochar alone.

## Figures and Tables

**Table 1:** The pH and elemental composition of lime and three biochars used in the experiment.

Materials	pH	C (%)	Si (%)	Al (%)	Fe (%)	Ca (%)	Mg (%)	K (%)	Na (%)	P (%)
Lime	12.88		1.34	0.28	0.14	52.4	0.81	0.04	-	0.01
SB	9.59	48.5	8.37	0.53	1.83	12.5	0.73	6.75	0.15	0.37
WB	9.92	59.2	5.22	1.27	3.62	7.05	0.30	1.81	0.08	0.30
MB	10.98	32.5	3.45	0.30	1.29	18.4	2.07	4.63	0.20	7.85

**Table 2:** The basic properties of the soils amended with biochar and lime. Values are means ± standard errors (*n* = 3).

Treatments	pH	Ex-Acid (cmol kg^−1^)	SOM (g kg^−1^)	CEC (cmol kg^−1^)	Total P (g kg^−1^)	Available K (mg kg^−1^)	MBC (mg kg^−1^)	MBN (mg kg^−1^)	MBP (mg kg^−1^)
CK	5.08 ± 0.07 e	1.05 ± 0.11 a	18.68 ± 0.32 d	11.46 ± 0.43 d	0.79 ± 0.04 c	66.84 ± 4.45 d	79.7 ± 12.68 e	12.77 ± 2.19 c	5.73 ± 0.37 e
Lime	5.49 ± 0.09 d	0.26 ± 0.05 c	17.96 ± 0.46 d	11.79 ± 0.13 cd	0.75 ± 0.03 c	67.73 ± 4.67 cd	81.94 ± 10.99 e	21.72 ± 1.47 c	7.14 ± 2.23 de
SB	5.53 ± 0.08 d	0.44 ± 0.10 b	31.03 ± 0.57 c	13.64 ± 0.55 b	0.99 ± 0.07 ab	114.54 ± 12.27 a	205.74 ± 18.18 d	69.87 ± 6.70 a	17.59 ± 2.20 b
WB	5.73 ± 0.08 c	0.43 ± 0.09 b	30.32 ± 1.09 c	12.03 ± 0.30 cd	0.89 ± 0.05 bc	90.36 ± 7.91 bc	193.11 ± 5.39 d	52.44 ± 5.48 b	11.47 ± 1.73 cd
MB	6.01 ± 0.06 b	0.41 ± 0.08 b	35.02 ± 1.52 b	13.89 ± 0.49 ab	0.99 ± 0.14 ab	109.27 ± 9.42 ab	279.8 ± 9.46 b	61.69 ± 5.96 ab	17.09 ± 1.21 b
SBL	6.04 ± 0.05 b	0.22 ± 0.03 c	33.87 ± 0.90 b	14.23 ± 0.29 ab	1.03 ± 0.07 ab	117.71 ± 12.00 a	244.09 ± 14.06 c	61.87 ± 7.79 ab	27.02 ± 3.96 a
WBL	5.77 ± 0.08 c	0.22 ± 0.03 c	31.34 ± 1.11 c	12.46 ± 0.17 c	1.06 ± 0.03 a	87.91 ± 8.74 c	290.50 ± 13.64 b	49.42 ± 2.03 b	15.77 ± 2.39 bc
MBL	6.32 ± 0.13 a	0.22 ± 0.03 c	36.91 ± 0.11 a	14.73 ± 0.77 a	1.08 ± 0.08 a	111.33 ± 10.01 a	347.47 ± 26.52 a	71.76 ± 7.79 a	30.65 ± 2.70 a
BT	30.19 ***	0.05 ^ns^	28.14 ***	21.79 ***	0.69 ^ns^	7.78 **	34.68 ***	8.02 **	19.29 ***
BL	34.85 ***	28.11 ***	11.29 **	5.29 *	5.01 *	0.03 ^ns^	53.89 ***	0.01 ^ns^	39.31 ***
BT×BL	7.57 **	0.04 ^ns^	0.84 ^ns^	0.19 ^ns^	0.61 ^ns^	0.09 ^ns^	3.40 ^ns^	2.22 ^ns^	23.40 ^ns^

Note: Ex-acid, exchangeable acid; SOM, soil organic matter; CEC, cation exchange capacity; MBC, microbial biomass C; MBN, microbial biomass N; MBP, microbial biomass P. BT, biochar type; BL, whether biochar was applied with lime. Different letters indicate significant differences between treatments (*p* < 0.05, LSD test). *—*p* < 0.05, **—*p* < 0.01, ***—*p* < 0.001, ns—not significant.

**Table 3:** Effects of biochar application alone and co-application with lime on soil enzyme activities. Values are means ± standard errors (*n* = 3).

Treatments	DHA (μg TPF g^−1^ h^−1^)	ACP (μg phenol g^−1^ h^−1^)	ALP (μg phenol g^−1^ h^−1^)	PD (μg phenol g^−1^ h^−1^)	IPP (μg PO_4_^3-^ g^−1^ h^−1^)
CK	275.21 ± 13.87 ab	228.26 ± 10.34 a	16.38 ± 2.83 f	111.90 ± 3.18 c	58.82 ± 5.45 d
Lime	254.64 ± 7.93 bc	164.64 ± 5.70 c	39.06 ± 5.16 e	91.37 ± 6.49 c	64.13 ± 4.12 d
SB	233.24 ± 9.75 c	193.72 ± 3.06 b	117.55 ± 10.88 b	146.54 ± 6.82 b	96.22 ± 5.45 c
WB	250.06 ± 6.60 bc	163.70 ± 5.33 c	76.84 ± 3.25 d	143.71 ± 6.87 b	81.11 ± 2.14 c
MB	196.32 ± 11.48 d	127.90 ± 3.57 e	117.14 ± 8.93 b	176.14 ± 18.67 a	114.76 ± 6.78 b
SBL	265.35 ± 7.61 ab	196.38 ± 2.26 b	98.12 ± 2.20 c	181.87 ± 21.18 a	122.17 ± 7.76 b
WBL	282.67 ± 15.54 a	146.71 ± 5.45 d	127.22 ± 13.08 b	144.96 ± 6.88 b	94.80 ± 4.84 c
MBL	143.77 ± 12.78 e	167.93 ± 6.00 c	148.45 ± 12.41 a	187.26 ± 19.61 a	165.01 ± 14.33 a
BT	178.23 ***	127.34 ***	11.91 **	6.34 *	44.28 ***
BL	0.92 ^ns^	10.89 **	14.40 **	3.44 ^ns^	43.74 ***
BT×BL	17.85 ***	41.49 ***	14.50 **	1.40 ^ns^	5.62 *

Note: DHA, dehydrogenase; ACP, acidic phosphomonoesterase; ALP, alkaline phosphomonoesterase; PD, phosphodiesterase; IPP, inorganic pyrophosphatase. BT, biochar type; BL, whether biochar was applied with lime. Different letters indicate significant differences between treatments (*p* < 0.05, LSD test). *—*p* < 0.05, **—*p* < 0.01, ***—*p* < 0.001, ns—not significant.

**Table 4:** Effects of biochar application alone and co-application with lime on the soil P functional genes (copies g^−1^). Values are means ± standard errors (*n* = 3).

Treatments	*phoC* (×10^7^)	*phoD* (×10^9^)	*gcd* (×10^8^)	*pqqC* (×10^7^)
CK	3.35 ± 0.27 a	0.39 ± 0.07 d	1.82 ± 0.05 d	4.85 ± 0.32 d
Lime	0.16 ± 0.06 e	0.47 ± 0.1 d	0.61 ± 0.12 e	1.04 ± 0.48 e
SB	3.17 ± 0.3 a	1.04 ± 0.1 c	3.05 ± 0.24 c	6.12 ± 0.18 cd
WB	2.35 ± 0.2 b	0.59 ± 0.23 d	2.95 ± 0.16 c	6.07 ± 0.17 cd
MB	2.44 ± 0.22 b	1.42 ± 0.12 bc	6.48 ± 0.32 a	12.05 ± 0.88 a
SBL	1.71 ± 0.2 c	2.34 ± 0.26 a	5.43 ± 0.9 b	8.55 ± 0.74 b
WBL	0.38 ± 0.06 e	1.78 ± 0.09 b	3.64 ± 0.1 c	6.33 ± 0.33 c
MBL	1.28 ± 0.09 d	2.47 ± 0.29 a	6.83 ± 0.79 a	12.49 ± 0.9 a
BT	29.71 ***	14.82 **	44.23 ***	108.54 ***
BL	179.50 ***	103.60 ***	14.36 **	8.51 *
BT×BL	4.36 *	0.38 ^ns^	4.30 *	3.77 ^ns^

Note: BT, biochar type; BL, whether biochar was applied with lime. Different letters indicate significant differences between treatments (*p* < 0.05, LSD test). *—*p* < 0.05, **—*p* < 0.01, ***—*p* < 0.001, ns—not significant.

## Data Availability

The original contributions presented in the study are included in the article, further inquiries can be directed to the corresponding author.
